# Supplementary material for: A Systematic Review and Meta-Analysis of Ocular and Periocular Basal Cell Carcinoma with First-Time Description of Dermoscopic and Reflectance Confocal Microscopy Features of Caruncle Basal Cell Carcinoma
Source: Diagnostics (Basel). 2025 May 14;15(10):1244. doi: 10.3390/diagnostics15101244 (PMC12109886; doi:10.3390/diagnostics15101244)
Supplement: Supplementary file 1 [file diagnostics-15-01244-s001.zip › Table S1 - List of excluded studies.docx]

| **Title** | **Authors** | **Exclusion criteria** |
| --- | --- | --- |
| Ocular basal cell carcinoma: a brief literature review of clinical diagnosis and treatment | Shi Y et al. (2017) | Review |
| Ocular sebaceous gland carcinoma: an update of the literature | Cicinelli MV and Kaliki S (2019) | Review |
| Cancers of the eye | Maheshwari A and Finger PT (2018) | Review |
| Xeroderma pigmentosum | Kraemer KH et al. (2023) | Review |
| Differential diagnosis of the swollen red eyelid | Carlisle RT and Digiovanni J (2015) | Review |
| BAP1 tumor predisposition syndrome | Pilarski R et al. (2016) | Review |
| Hermansky-Pudlak syndrome | Introne WJ et al. (2000) | Review |
| Current diagnostics and therapy recommendations for ocular basal cell carcinoma | Kakkassery V et al. (2017) | German |
| Management of periorbital basal cell carcinoma with orbital invasion | Sun MT et al. (2015) | Review |
| Nevoid basal cell carcinoma syndrome: a case report and literature review | Chitta S et al. (2022) | Review |
| Vismodegib (ERIVEDGE°) in basal cell carcinoma: too many unknowns | No authors | No full text |
| Immunohistochemical distinction of ocular sebaceous carcinoma from basal cell and squamous cell carcinoma | Sinard JH (1999) | No ocular BCC |
| Eyelid and ocular surface carcinoma: diagnosis and management | Yin VT et al. (2015) | Review |
| Basal cell carcinoma of the eyelids | Shields CL (1993) | Review |
| Nevoid basal cell carcinoma syndrome (Gorlin syndrome) | Lo Muzio L (2008) | Review |
| Ocular dermoid in patient with basal cell nevus syndrome | Shah M and Hanke W (2020) | No ocular BCC |
| Sebaceous carcinoma of the ocular adnexa | Kass LG and Hornblass A (1989) | Review |
| Xeroderma pigmentosum | Zghal M et al. (2018) | French |
| Health risks | Longstreth J et al. (1998) | Review |
| Recognizing basal cell carcinoma on smartphone-captured digital histopathology images with a deep neural network | Jiang YQ et al. (2020) | No ocular BCC |
| Basal cell carcinomas of the eyelids | Allali J et al. (2005) | Review |
| Basal cell nevus syndrome: clinical and molecular review and case report | Pino LC et al. (2016) | Review |
| Electrophysiological study of visual pathways in nevoid basal cell carcinoma syndrome patients | Moramarco A et al. (2021) | Ocular manifestation in nevoid basal cell carcinoma syndrome |
| Understanding nevoid basal cell carcinoma syndrome (Gorlin syndrome): a case report | Olivero Y (2023) | No ocular BCC |
| Emerging therapeutic options for periorbital and orbital cutaneous basal and squamous cell carcinomas | Wladis EJ et al. (2023) | Review |
| Human papillomavirus related neoplasia of the ocular adnexa | Ramberg I and Heegaard S (2021) | Review |
| Oculoplastic aspects of ocular oncology | Rene C (2013) | Review |
| Pigmented squamous cell carcinoma | Chapman MS et al. (2000) | SCC |
| Therapy failure and resistance mechanism in eyelid and ocular surface tumors | Ju X et al. (2022) | Review |
| Basal cell carcinoma of the eyelid as an indicator of multifocal malignancy | Wesley RE (1982) | No ocular BCC |
| Immunohistochemistry in ocular carcinomas | Sramek B (2008) | No ocular BCC |
| The role of surgery after remission of nonsystemic extensive periorbital basal cell carcinoma treated by Vismodegib: a systematic review | Peillex D et al. (2022) | Review |
| Techniques and applications in skin OCT analysis | Yow AP et al. (2020) | Review |
| Current therapies in superficial malignant tumors | Scholz SL et al. (2019) | German |
| Optimizing the effective doses of mitomycin C, 5-fluorouracil, and their combination on cultivated basal cell carcinoma | Balagholi S et al. (2021) | No ocular BCC |
| Epidemiology of cutaneous sebaceous carcinoma | Wu A et al. (2021) | Sebaceous carcinoma |
| Primary periocular squamous cell carcinoma in central Spain: factors related to recurrence | Galindo-Ferreiro A et al. (2022) | Periocular SCC |
| Netarsudil-related eyelid wound dehiscence | Kim HM et al. (2021) | No full text |
| Ultraviolet light and ocular diseases | Yam JC and Kwok AK (2014) | Review |
| Current therapies in superficial malignant tumors | Scholz SL et al. (2018) | German |
| Xeroderma pigmentosum | Stary A and Sarasin A (1997) | French |
| Ocular manifestations in Gorlin-Goltz syndrome | Moramarco A et al. (2019) | No ocular BCC |
| Radiation therapy: conjunctival and eyelid tumors | Aronow ME and Singh AD (2013) | Review |
| Orbital exenteration | Benazzou S et al. (2011) | French |
| Primary basosquamous carcinoma of the lower eyelid with ocular invasion: a case report | Papadopoulos GO et al. (2018) | Basosquamous tumor |
| Pigmented squamous cell carcinoma: case report and importance of differential diagnosis | Morais PM et al. (2018) | SCC |
| A child with xeroderma pigmentosum for excision of basal cell carcinoma | Mulimani SM and Talikoti DG (2013) | No ocular BCC |
| Clinical testing for the nevoid basal cell carcinoma syndrome in a DNA diagnostic laboratory | Klein RD et al. (2005) | Nevoid basal cell carcinoma syndrome |
| Pediatric genetic ocular tumors | Rouhani B and Ramasubramanian A (2014) | No ocular BCC |
| Basal cell nevus syndrome presenting as epiretinal membrane and myelinated nerve fiber layer | Farley ND et al. (2017) | Nevoid basal cell carcinoma syndrome |
| Syringocystadenocarcinoma papilliferum of the eyelid | Hoguet AS et al. (2012) | Review |
| FOXC1, the new player in the cancer sandbox | Elian FA et al. (2017) | Review |
| Supraestructure maxillectomy and orbital exenteration for treatment of basal cell carcinoma of inferior eyelid: case report and review | Sebastián Villalón-López J. et al. (2005) | No full text |
| Acquired lacrimal sac fistula mimicking basal cell carcinoma | Litwin AS et al. (2013) | Fistula |
| Sebaceous gland carcinoma of the ocular adnexa—variability in clinical and histological appearance with analysis of immunohistochemical staining patterns | Schmitz EJ et al. (2017) | Sebaceous gland carcinoma |
| Ocular manifestations of genetic skin disorders | Jen M and Nallasamy S. (2016) | Review |
| Malignant lesions of the eyelids | Piest KL (1992) | Review |
| KLF4 coordinates corneal epithelial apical-basal polarity and plane of cell division and is downregulated in ocular surface squamous neoplasia | Tiwari A et al. (2020) | No ocular BCC |
| What size of surgical margins for carcinoma of the eyelid? | Mouriaux F et al. (2015) | French |
| Intratarsal keratinous eyelid cysts in Gorlin syndrome: a review and reappraisal | Wolkow N et al. (2018) | Review |
| A histochemical and immunohistochemical study of extra-ocular sebaceous carcinoma | Ansai S et al. (1993) | Sebaceous carcinoma |
| S100A expression in normal corneal-limbal epithelial cells and ocular surface squamous cell carcinoma tissue | Li J et al. (2011) | SCC |
| Pathophysiology of ocular surface squamous neoplasia | Gichuhi S et al. (2014) | Review |
| Incompletely excised basal cell carcinoma of the ocular adnexa | Wiggs EO (1981) | No full text |
| Can the onset of orbital cancer be the result of a prosthetic eye? | Croce A et al. (2017) | Review |
| Epidemiological evidence of carcinogenicity of sunbed use and of efficacy of preventive measures | Gandini S et al. (2019) | Review |
| Interventional radiotherapy (brachytherapy) in eyelid and ocular surface tumors: a review for treatment of naïve and recurrent malignancies | Pagliara MM et al. (2022) | Review |
| Visual diagnosis of hematologic and oncologic diseases | Blackburn P (1993) | No full text |
| Orbital exenteration for invasive skin tumours | Tyers AG (2006) | Review |
| Primary cutaneous histoplasmosis mimicking basal cell carcinoma of the eyelid: a case report and review of literature | Radhakrishnan S (2016) | Histoplasmosis |
| An immunohistochemical study of BCA-225 in various skin cancers | Ansai S et al. (1994) | Missing data |
| The spectrum of oculocutaneous disease: Part II. Neoplastic and drug-related causes of oculocutaneous disease | Day A et al. (2014) | Review |
| Xeroderma pigmentosum in Libya | Khatri ML et al. (1999) | No ocular BCC |
| Two unique BAP1 pathogenic variants identified in the same family by panel cascade testing | Byrne L et al. (2022) | No full text |
| Squamous cell carcinoma of the eyelid treated with photodynamic therapy | Rossi R et al. (2004) | SCC |
| Nevoid basal cell carcinoma syndrome | McNamara RJ (1976) | Nevoid basal cell carcinoma syndrome |
| Editorial: Hot topics in ocular pharmacology | Grzybowski A (2017) | Ocular pharmacology |
| Observational prospective cohort study of patients with newly-diagnosed ocular sebaceous carcinoma | Muqit MM et al. (2013) | Sebaceous carcinoma |
| Ocular sebaceous carcinoma. Two unusual cases, and their histochemical and immunohistochemical findings | Sugiki H et al. (1996) | Sebaceous carcinoma |
| A clinicopathological classification analysis of ocular mass lesions in 7 910 cases | Wang LY et al. (2019) | Chinese |
| Pigmented squamous cell carcinoma in situ of the conjunctiva in 5 cases | Shields CL et al. (2008) | SCC |
| Nevoid basal-cell syndrome: literature review and case report in a family | José Tincani A et al. (1995) | Review |
| Multifocal metastasizing extra-ocular facial sebaceous carcinoma as diagnostic challenge: case report and systematic review | Bolm I et al. (2015) | Sebaceous carcinoma |
| Serum malondialdehyde as a biomarker of oxidative stress in patients with primary ocular carcinoma: impact on response to chemotherapy | Maurya RP et al. (2021) | Level of serum malondialdehyde |
| Adverse effects of ultraviolet radiation: a brief review | Gallagher RP and Lee TK (2006) | Review |
| Changes in ultraviolet radiation exposure to the ocular region: a population-based study | Weis E et al. (2019) | No ocular BCC |
| Trichoadenoma of the eyelid | Shields JA et al. (1998) | Trichoadenoma of the eyelid |
| Liability for the treatment of anterior segment eye disease | Classé JG (1991) | No full text |
| Sebaceous carcinoma: an immunohistochemical reappraisal | Ansai S et al. (2011) | Sebaceous carcinoma |
| Xeroderma pigmentosum in black South Africans | Jacyk WK (1999) | Review |
| Periocular malignancies and primary eye care | Myers M and Gurwood AS (2001) | No full text |
| Trichoblastic fibroma of the eyelid | Wladis EJ et al. (2012) | Trichoblastic fibroma of the eyelid |
| Biochemical aspects of ocular tumors. Content of Na, K, Ca, Mg, P, and Cu in retinoblastoma and invasive basal cell carcinoma of the conjunctiva and values of these minerals in the eye tissues | De Jorge FB et al. (1969) | Portuguese |
| Supraestructure maxillectomy and orbital exenteration for treatment of basal cell carcinoma of inferior eyelid: case report and review | Villalon-Lopez JS et al. (2006) | No full text |
| Ocular hypertelorism, facial basal cell carcinomas, and multiple odontogenic keratocysts of the jaws | Friedlander AH et al. (1988) | No ocular BCC |
| Oculoplastic surgeries in patients older than 90 years of age | Abumanhal M et al. (2022) | No full text |
| A rare case of Gorlin-Goltz syndrome in children | Boos Lima FBDJ et al. (2019) | No ocular BCC |
| Margin eyelid reconstruction with hard palate graft | Burmann TG et al. (2008) | Portuguese |
| Sebaceous cell carcinomas of the ocular adnexa | Ni C et al. (1982) | Sebaceous cell carcinomas |
| Malignant lesions of the eyelid | Mamalis N et al. (1989) | Review |
| Basal cell nevus syndrome: guidelines for early detection | Bitar GJ et al. (2002) | Basal cell nevus syndrome |
| Cutaneous horn of the eyelid in 13 cases | Pointdujour-Lim R et al. (2017) | No ocular BCC |
| The human health effects of ozone depletion and interactions with climate change | Norval M et al. (2011) | Review |
| Periocular reconstruction: a systematic approach | Spinelli HM and Jelks GW (1993) | No full text |
| Mohs micrographic surgery of the eyelid: an overview of anatomy, pathophysiology, and reconstruction options | Harvey DT et al. (2013) | Review |
| Ocular manifestations in xeroderma pigmentosum | Süsskind D et al. (2011) | German |
| Interstitial HDR brachytherapy in the treatment of non-melanocytic skin cancers around the eye | Cisek P et al. (2021) | Missing data |
| Epidemiology of ocular surface squamous neoplasia in veterans: a retrospective case-control study | Smith LM et al. (2019) | No ocular BCC |
| Primary cutaneous angiosarcoma of the eyelid: a diagnostic and therapeutic challenge | Milman T et al. (2018) | Angiosarcoma |
| Gorlin's syndrome. Case report | Salati C et al. (1997) | No ocular BCC |
| Analysis of hedgehog signaling in periocular sebaceous carcinoma | Bladen JC et al. (2018) | Sebaceous cell carcinomas |
| Plaque radiotherapy in the management of scleral-invasive conjunctival squamous cell carcinoma: an analysis of 15 eyes | Arepalli S et al. (2014) | SSC |
| Recurrence rates of treated basal cell carcinomas. Part 2: Curettage-electrodesiccation | Silverman MK et al. (1991) | No ocular BCC |
| Cutaneous leishmaniasis of the eyelids: a case series with molecular identification and literature review | Mohammadpour I et al. (2016) | Review |
| Mucin-producing malignant tumor of lower eyelid presenting in a 14-year-old patient | Latorre A et al. (2012) | Mucin-producing malignant tumor |
| Epidemiology of ocular surface squamous neoplasia in a Veterans Affairs population | McClellan AJ et al. (2013) | Ocular surface squamous neoplasia |
| Rippled pattern extraocular sebaceous carcinoma: a rare case report with brief review of literature | Amita K et al. (2013) | Sebaceous carcinomas |
| Oronasal squamous cell carcinomas in françois’ langurs (trachypithecus francoisi) | Flanders JA et al. (2017) | Trachypithecus francoisi |
| Basal cell epithelioma of the eyelids | Bonvallot T et al. (1988) | French |
| Ocular metastasis of cutaneous malignant melanoma | Ullah T et al. (2009) | Metastasis |
| Xeroderma pigmentosum at a tertiary care center in Saudi Arabia | Alwatban L and Binamer Y (2017) | No ocular BCC |
| Ocular tumors | Haye C and Dufier JL (1975) | French |
| Darier disease mimicking basal cell carcinoma of the eyelid | Russell DJ et al. (2009) | No ocular BCC |
| Mucoepidermoid carcinoma of eyelid: a usual tumor at an unusual site | Singh L et al. (2015) | Mucoepidermoid carcinoma of the eyelid |
| Elevated expression of ABCB5 in ocular surface squamous neoplasia | Jongkhajornpong P (2016) | No ocular BCC |
| Sebaceous carcinoma: clinicopathologic features and diagnostic role of immunohistochemistry (including androgen receptor) | Mulay K et al. (2014) | SCC |
| Ocular myiasis in a glioma: a case report | Mathew DJ et al. (2016) | Glioma |
| Extraocular sebaceous carcinoma on the chest wall: a case report | Mn R et al. (2014) | Sebaceous carcinoma |
| Expression pattern of sonic hedgehog and effect of topical mitomycin C on its expression in human ocular surface neoplasms | Fujita K et al. (2008) | SCC |
| Protection of the eye from ultraviolet radiation damage among adults in Addis Zemen Town, Northwest Ethiopia | Belete GT et al. (2021) | No ocular BCC |
| Ocular surface squamous neoplasia: outcomes following primary excision with 2 mm margin and cryotherapy | Bowen RC et al. (2021) | Ocular surface squamous neoplasia |
| Epigenetic inactivation of the E-cadherin gene in eyelid sebaceous gland carcinoma | Jayaraj P et al. (2012) | No ocular BCC |
| Myths in the diagnosis and management of orbital tumors | Gündüz K and Yanık Ö (2015) | No ocular BCC |
| Gorlin-Goltz syndrome: a case series of 5 patients in North Indian population with comparative analysis of literature | Lata J (2015) | No ocular BCC |
| Expression of SIRT1 in ocular surface squamous neoplasia | Alves LF et al. (2012) | Ocular surface squamous neoplasia |
| Cytologic evaluation of ocular lesions | Sanderson TL et al. (1980) | No full text |
| Solitary trichoepithelioma of the eyelid: a clinico-pathological correlation | Gupta A et al. (2015) | Trichoepithelioma |
| Pilomatrix carcinoma of the lacrimal caruncle: a case report | Harbiyeli II et al. (2020) | Pilomatrix carcinoma |
| Exploration of potential key pathways and genes in multiple ocular cancers through bioinformatics analysis | Wan Q and Tang J (2019) | Missing data |
| Cutaneous sebaceous carcinoma presenting as a large fungating breast tumour in synchronicity with primary carcinomata of the breasts | Makaranka S et al. (2022) | Sebaceous carcinoma |
| Cytokeratin 5/6 immunostaining in cutaneous adnexal neoplasms and metastatic adenocarcinoma | Plumb SJ et al. (2004) | Distribution of CK 5/6 in cutaneous neoplasm |
| Cluster headache after orbital exenteration | Bond JB and Wesley RE (1987) | No full text |
| Mutations in exon 3 of the CTNNB1 gene (beta-catenin gene) in cutaneous adnexal tumors | Kazakov DV et al. (2009) | No ocular BCC |
| Preoperative brush and impression cytology in ocular surface squamous neoplasms | Ersöz C et al. (2003) | Squamous neoplasms |
| Xeroderma pigmentosum. Cutaneous, ocular, and neurologic abnormalities in 830 published cases | Kraemer KH et al. (1987) | No full text |
| Epidemiological and clinical characteristics of primary ocular cancers in blacks: our experience with 111 cases | Seck SM et al. (2015) | French |
| Ocular surface squamous neoplasia as the initial presenting sign of human immunodeficiency virus infection in 60 Asian Indian patients | Kaliki S et al. (2017) | Ocular surface squamous neoplasia |
| Immunohistochemical findings of sebaceous carcinoma and sebaceoma: retrieval of cytokeratin expression by a panel of anti-cytokeratin monoclonal antibodies | Ansai S et al. (2011) | Sebaceous carcinoma and sebaceoma |
| Clinical observation of immunotherapy in ocular malignant tumors with lymphokine-activated killing (LAK) cell | Yang L et al. (1993) | Chinese |
| Regression of sebaceous carcinoma of the eyelid after a small incisional biopsy: report of two cases | Brownstein S et al. (2019) | Sebaceous carcinoma |
| Clinicopathological report: mucinous carcinoma of the eyelid | Lee GA et al. (1999) | Mucinous carcinoma |
| LRIG1 as a potential novel marker for neoplastic transformation in ocular surface squamous neoplasia | Nagata M et al. (2014) | No ocular BCC |
| Papillomatosis of conjunctiva and adnexa in dogs | Bonney CH et al. (1980) | No full text |
| Sebaceoma and related neoplasms with sebaceous differentiation: a clinicopathologic study of 30 cases | Misago N et al. (2002) | Sebaceoma |
| Dimorphic immunohistochemical staining in ocular sebaceous neoplasms: a useful diagnostic aid | Johnson JS et al. (1999) | Sebaceous carcinoma |
| Exenteration and adjuvant radiotherapy for primary carcinosarcoma of the anterior orbit: a case report | Fernandez C et al. (2021) | Carcinosarcoma |
| Orbital exenteration with eyelid sparing: indications, technique, and results | Shields JA et al. (1991) | No full text |
| Perforin expression in eyelid sebaceous carcinomas: a useful and specific immunomarker for the differential diagnosis of eyelid carcinomas | Mittal R et al. (2016) | Sebaceous carcinoma |
| Interferon Alfa-2b for pigmented ocular surface squamous neoplasia: a report of 8 lesions | Kaliki S et al. (2021) | Squamous neoplasms |
| Simultaneous adenomatoid odontogenic and keratocystic odontogenic tumours in a patient with Gorlin-Goltz syndrome | Shephard M and Coleman H (2014) | No ocular BCC |
| Primary treatment of ocular surface squamous neoplasia with topical interferon alpha-2b: comparative analysis of outcomes based on original tumor configuration | Shields CL et al. (2021) | Squamous neoplasms |
| Upregulated MYC expression and p53 mutations may contribute to the oncogenesis of canine Meibomian gland carcinomas | Peterson C et al. (2023) | Meibomian gland carcinomas |
| Cutaneous neoplasms in dogs associated with canine oral papillomavirus vaccine | Bregman CL et al. (1987) | Cutaneous neoplasms in dogs |
| Basaloid follicular hamartoma of the eyelid: a case report and literature review about an unusual lesion in the ocular region | Lamas NJ et al. (2022) | Basaloid follicular hamartoma |
| Outcome of patients with periocular sebaceous gland carcinoma with and without conjunctival intraepithelial invasion | Chao AN et al. (2001) | Sebaceous cell carcinomas |
| Ocular surface squamous neoplasia with intraocular tumour extension: a study of 23 patients | Kaliki S et al. (2020) | Ocular surface squamous neoplasia |
| The eye in malignant disease | Lau CH and Taylor S (2003) | Review |
| The Gorlin-Goltz syndrome: case report | Manzi G et al. (1990) | No ocular BCC |
| Role of Köllner-Hughes tarsoconjunctival flap in the reconstruction of large eyelid defects | Bouazza M et al. (2017) | French |
| Blepharopigmentation and eyebrow enhancement techniques for maximum cosmetic results | Angres GG (1985) | No full text |
| Bilateral aggressive basal cell carcinoma of the eyelids and face—case report | Włodarkiewicz A et al. (1998) | Polish |
| Ocular malignant tumors. Review of the Tumor Registry at a tertiary eye hospital in central Saudi Arabia | Khandekar RB et al. (2014) | No full text |
| Use of the autologous dorsal dermis in reconstruction of the posterior palpebral lamella in blepharopoiesis | Azoulay L et al. (2019) | French |
| Multiple keratocysts of the mandible in association with Gorlin-Goltz syndrome: a rare case report | Kulkarni GH et al. (2014) | No ocular BCC |
| Syndromic odontogenic keratocyst: a case report and review of literature | Arshad F (2016) | No ocular BCC |
| Anterior segment optical coherence tomography features of pseudoepitheliomatous hyperplasia of the ocular surface: a study of 9 lesions | Kaliki S (2021) | Pseudoepitheliomatous hyperplasia |
| Histopathology-guided management of ocular surface squamous neoplasia with corneal stromal or scleral invasion using ruthenium-106 plaque brachytherapy | Rao R et al. (2023) | Squamous neoplasia |
| Alteration of expression pattern of transient receptor potential vanilloid 2 and transient receptor potential vanilloid 3 in ocular surface neoplasm | Izutani-Kitano A et al. (2020) | No ocular BCC |
| Sebaceous carcinoma of the face following irradiation | Hood IC et al. (1986) | Sebaceous carcinoma |
| Reconstruction of the levator function and conjunctiva using an inferiorly based orbital septal flap for subtotal full-thickness defects of the upper eyelid: two cases of ocular sebaceous carcinoma | Ohshiro T et al. (2006) | Sebaceous carcinoma |
| The effects on human health from stratospheric ozone depletion and its interactions with climate change | Norval M et al. (2007) | No ocular BCC |
| Expression pattern of the bullous pemphigoid-180 antigen in normal and neoplastic epithelia | Fairley JA et al. (1995) | No ocular BCC |
| Ocular lens blue autofluorescence cannot be used as a measure of individual cumulative UVR exposure | Sandby-Møller J et al. (2004) | No ocular BCC |
| Benign ocular adnexal tumours of apocrine, eccrine or hair follicle origin | Ozdal PC et al. (2003) | Benign ocular adnexal tumors |
| Giant ocular surface squamous neoplasia managed with interferon alpha-2b as immunotherapy or immunoreduction | Kim HJ et al. (2012) | Ocular surface squamous neoplasia |
| Extending far and wide: the role of biopsy and staging in the management of ocular surface squamous neoplasia | Polski A et al. (2019) | Ocular surface squamous neoplasia |
| Epithelial tumors of the corneal limbus | Cernea P et al. (1994) | Romanian |
| Ocular surface squamous neoplasia: analysis based on the 8th American Joint Committee on Cancer classification | Singh S et al. (2019) | Ocular surface squamous neoplasia |
| Genetic study of familial uveal melanoma: association of uveal and cutaneous melanoma with cutaneous and ocular nevi | Smith JH et al. (2007) | No ocular BCC |
| Cutaneous and ocular side-effects of oral photochemotherapy: results of an 8-year follow-up study | Cox NH et al. (1987) | No ocular BCC |
| Treatment of cancer in a child with ocular xeroderma pigmentosa (XP) in Malawi | Schulze Schwering M et al. (2014) | SSC |
| Cytology of ocular lesions | Naib ZM (1972) | No full text |
| The role of cryosurgery in external ocular and periocular disease | Fraunfelder FT et al. (1977) | No full text |
| Concomitant simple limbal epithelial transplantation after surgical excision of ocular surface squamous neoplasia | Kaliki S et al. (2017) | OSSN |
| Skin cancers in patients of skin phototype V or VI with xeroderma pigmentosum type C (XP-C): a retrospective study | Ventéjou S et al. (2019) | No ocular BCC |
| Ultraviolet radiation and the role of matrix metalloproteinases in the pathogenesis of ocular surface squamous neoplasia | Ng J et al. (2008) | Ocular surface squamous neoplasia |
| Uveal metastasis from lung cancer: clinical features, treatment, and outcome in 194 patients | Shah SU et al. (2014) | No ocular BCC |
| Ocular findings in linear sebaceous naevus syndrome | Insler MS and Davlin L (1987) | No ocular BCC |
| Cutaneous eyelid tuberculosis—a case report | Wyrwicka A et al. (2011) | Polish |
| The expression of class I major histocompatibility antigens by human retinal pigment epithelium in vitro | Benson MT et al. (1992) | No ocular BCC |
| Spatial analysis of p63, K5 and K7 defines two groups of progenitor cells that differentially contribute to the maintenance of normal sebaceous glands, extraocular sebaceous carcinoma and benign sebaceous tumors | Boecker W et al. (2019) | Sebaceous carcinoma |
| High-resolution anterior segment optical coherence tomography in intraepithelial versus invasive ocular surface squamous neoplasia | Singh S et al. (2018) | OSSN |
| HMG-CoA reductase expression in human eyelid tissue and in a human meibomian gland epithelial cell line | Ooi KG et al. (2019) | No ocular BCC |
| Ocular malignancies of xeroderma pigmentosum: clinical and therapeutic features | Touzri RA et al. (2008) | French |
| Recombinant interferon alpha 2b for ocular surface squamous neoplasia: an efficient and cost-effective treatment modality in Asian Indian patients | Kaliki S et al. (2016) | Ocular surface squamous neoplasia |
| Tegaderm transparent dressing (3M) for the treatment of chronic exposure keratopathy | Airiani S et al. (2003) | No full text |
| Eyelid reconstruction with Fricke's flap: report of two cases | Herzog Neto G et al. (2006) | Portuguese |
| Bilateral epiretinal membranes in Gorlin syndrome associated with a novel PTCH mutation | Scott A et al. (2007) | No ocular BCC |
| Characterization of intraocular tumors arising in transgenic mice | Anand R et al. (1994) | No full text |
| Orbito-ocular tumors in Nigeria | Olurin O and Williams AO (1972) | No ocular BCC |
| Nasal reconstruction based on aesthetic subunits in Orientals | Yotsuyanagi T et al. (2000) | No ocular BCC |
| Familial odontogenic keratocysts. Report of 3 cases and review of Japanese dental literature | Kamiya Y et al. (1985) | No ocular BCC |
| Ocular manifestations of xeroderma pigmentosum | Khanna VN et al. (1965) | No full text |
| Gorlin syndrome: the PTCH gene links ocular developmental defects and tumour formation | Ragge NK et al. (2005) | No ocular BCC |
| The use of latex biomembrane in exenteration: case report | Fugimoto F et al. (2007) | Portuguese |
| A ten-year hospital survey of eyelid cancer | Francis IC et al. (1984) | No full text |
| Identification of a novel mutation in the PTCH gene in a patient with Gorlin-Goltz syndrome with unusual ocular disorders | Romano M et al. (2011) | No ocular BCC |
| Photo essay: combined hamartoma of the retina and retinal pigment epithelium in Gorlin syndrome | De Potter P et al. (2000) | No full text |
| Cutaneous and ocular changes associated with the use of chlorpromazine | Wolf ME et al. (1993) | No full text |
| A recurrent adenoid-type basal-cell epithelioma of the superior portion of the ala nasi and of the left internal palpebral angle (rodent ulcer)—a clinical case | Cârstocea B et al. (1998) | Romanian |
| Orbital amelanotic melanoma in xeroderma pigmentosum: a rare association | Rizvi S et al. (2008) | No ocular BCC |
| Orbit evisceration: a retrospective study on a consecutive series of 10 years | Szabo I et al. (2013) | Romanian |
| Adnexal carcinomas of the skin. II. Extraocular sebaceous carcinomas | Wick MR et al. (1985) | Sebaceous carcinoma |
| The surgical approach to invasive orbital tumors | Cernea P and Stefănescu A (1993) | Romanian |
| Xeroderma pigmentosa with ocular involvement (two case reports with discussion on the subject) | Sarma CC et al. (1973) | No full text |
| Laser surgery of ocular adnexa | Krasnov MM et al. (1983) | Russian |
| Combined surgery and cryotherapy for scleral invasion of epithelial malignancies | Dutton JJ et al. (1984) | No full text |
| Tumors and tumor-like lesions of eyelids collected at Department of Pathological Anatomy, Wroclaw Medical University, between 1946 and 1999 | Hałoń A et al. (2005) | Polish |
| Antiangiogenic treatment enhances photodynamic therapy responsiveness in a mouse mammary carcinoma | Ferrario A et al. (2000) | No full text |
| Prognosis of basalioma in the ocular region | Brewitt H et al. (1976) | German |
| Conjunctival epithelial carcinoma in a 9-year-old child with xeroderma pigmentosum. Case report | Rouberol F et al. (2001) | French |
| Cancer excision from eyelids and ocular adnexa. The Mohs' fresh tissue technique and reconstruction. A five-year study of 109 patients | Callahan MA et al. (1983) | No full text |
| Value of radiotherapy alone in basalioma of the ocular area | Gornig H and Beyer W (1969) | German |
| Curietherapy of palpebral epithelioma with iridium-192. Method and results apropos of 192 cases treated at the Claudius-Regaud Center | De Lafontan B et al. (1986) | French |
| Endocurietherapytechnic for epithelioma of ocular area (author’s transl) | Bolla M et al. (1980) | French |
| Ocular manifestations of xeroderma pigmentosum (report of 5 cases) | Ben Rayana N et al. (1998) | French |
| Long-term curative effect of cryotherapy for malignant ocular tumours | Zhang FK and Shi XR (1993) | Chinese |
| Ocular side effects of added sympathomimetics in local anesthetics | Häberle M and Fischer H (1990) | German |
| Pilomatrix carcinoma of the lacrimal caruncle: a case report | Harbiyeli II et al. (2019) | Pilomatrix carcinoma |
| Bilateral lacrimal system involvement by sclerosing extramedullary hematopoietic tumor | Ghazi NG et al. (2006) | No ocular BCC |
| Caruncular lesions: a 16-year single centre review in the United Kingdom | Yvon C et al. (2022) | No full text |
| Sebaceous carcinoma of the eyelid, eyebrow, caruncle and orbit | Boniuk M and Zimmerman LE (1972) | No full text |
| Neoplasms of the caruncle. Clinicopathologic study of 40 cases | Perucho-Martínez S et al. (2004) | Spanish |
| Primary basal cell carcinoma of the caruncle | Mihailovic N et al. (2019) | German |
| Malignant lesions of the caruncle | Gounder P et al. (2023) | No full text |
| Sebaceous carcinoma of the eyelids: personal experience with 60 cases | Shields JA et al. (2004) | Sebaceous carcinoma |
| Observational prospective cohort study of patients with newly-diagnosed ocular sebaceous carcinoma | Muqit MM et al. (2013) | Sebaceous carcinoma |
| Ocular surface squamous neoplasia: analysis based on the 8th American Joint Committee on Cancer classification | Singh S et al. (2019) | Ocular surface squamous neoplasia |
| Neoplasms of the caruncle. Clinicopathologic study of 40 cases | Perucho-Martínez S et al. (2004) | Spanish |
| HMG-CoA reductase expression in human eyelid tissue and in a human meibomian gland epithelial cell line | Ooi KG et al. (2019) | No ocular BCC |
| Periocular basal cell carcinoma | Heindl LM (2020) | German |
| Periocular basal cell carcinoma—clinical perspectives | Furdova A et al. (2020) | Review |
| Basal cell carcinoma of the eyelid and periocular skin | Margo CE and Waltz K (1993) | Review |
| Role of Vismodegib in the management of advanced periocular basal cell carcinoma | Cox KF and Margo CE (2016) | Review |
| Periocular basal cell carcinoma | González F and García A (2005) | Spanish |
| Periocular poroma—a rare differential diagnosis to basal cell carcinoma | Kesper C et al. (2022) | German |
| Surgical resection with ophthalmoplastic reconstruction: gold standard in periocular basal cell carcinoma | Rokohl AC et al. (2020) | German |
| Advanced periocular basal cell carcinoma—a therapeutic challenge | Lauterbach B et al. (2019) | German |
| Merkel cell carcinoma of the eyelid and periocular region: a review | Walsh NM (2022) | Merkel cell carcinoma |
| Hedgehog pathway inhibitors for periocular basal cell carcinoma | Ashraf DC and Vagefi MR (2020) | Missing data |
| Mohs micrographic surgery versus surgical excision for periocular basal cell carcinoma | Narayanan K et al. (2014) | Review |
| Periocular basal cell carcinoma recurrence following surgical treatment: safe surveillance time | Juniat V et al. (2023) | No full text |
| Periocular pigmented basal cell carcinomas: clinicopathologic features and mutational profile | Hassanin F et al. (2022) | No full text |
| A new paradigm in the treatment of advanced periocular basal cell carcinoma? | Unsworth SP et al. (2019) | Missing data |
| Mohs micrographic surgery versus surgical excision for periocular basal cell carcinoma | Narayanan K et al. (2009) | Review |
| Diagnosis of periocular basal cell carcinoma with optical coherence tomography | Adan F et al. (2021) | No full text |
| Comment on: “Periocular basal cell carcinoma in under 40s—Is more aggressive treatment warranted?” | Rajput R et al. (2020) | Missing data |
| Periocular basal cell carcinoma in under 40s—Is more aggressive treatment warranted? | Saxby E et al. (2019) | Missing data |
| Vismodegib for treatment of periocular basal cell carcinoma—6-year experience from a tertiary cancer center | Xavier C et al. (2021) | Missing data |
| Guidelines for Vismodegib in the management of periocular basal cell carcinoma | Hussain A et al. (2020) | Missing data |
| Growth of periocular basal cell carcinoma | Rademaker M (2015) | Missing data |
| Merkel cell carcinoma of the eyelid: a review | North VS et al. (2019) | Merkel cell carcinoma |
| Periocular basal cell carcinoma treatment tendencies | García Martín E et al. (2010) | Spanish |
| Basal cell carcinoma of the periocular region | Geszti F et al. (2013) | German |
| Healthcare resource utilization and cost of care in patients with periocular basal cell carcinoma: a real-world study | Kahana A et al. (2022) | Missing data |
| Recurrence rates of basal cell carcinoma of the periocular skin: what to do with patients who have positive margins after resection | Jebodhsingh KN et al. (2012) | Missing data |
| The effect of socio-economic status on severity of periocular basal cell carcinoma at presentation | Lim LT et al. (2015) | Missing data |
| Recurrence risk of periocular basal cell carcinoma after histologically controlled excision | Dethmers A et al. (2023) | German |
| Malignant eyelid lesions | Rana H et al. (2022) | Missing data |
| Eyelid basal cell carcinoma with neuroendocrine differentiation: a case report and literature review | Chung IY et al. (2021) | Basosquamous tumor |
| Vismodegib improves quality of life in patients with periocular locally advanced basal cell carcinoma: subgroup analysis, STEVIE trial | Gershoni A et al. (2022) | Missing data |
| Treatment of periocular basal cell carcinoma using an advanced stereotactic device | Pontoriero A et al. (2014) | No full text |
| Basal cell carcinoma: a single-center experience | Abbas OL and Borman H (2012) | Missing data |
| Management of periocular basal cell carcinoma: Mohs’ micrographic surgery versus radiotherapy | Leshin B et al. (1993) | Missing data |
| SOP—Standardized procedures in diagnostics and therapies of periocular basal cell carcinoma | Kakkassery V and Heindl LM (2017) | German |
| PCR quantification of *D. folliculorum* in periocular basal cell carcinoma | Sánchez España JC et al. (2016) | Missing data |
| Letter to the editor: Effective treatment of locally advanced periocular basal cell carcinoma with oral hedgehog pathway inhibitor | Yuksel N (2021) | Missing data |
| Alternative treatment options for periorbital basal cell carcinoma | Kakkassery V et al. (2020) | German |
| Vismodegib as eye-sparing neoadjuvant treatment for locally advanced periocular basal cell carcinoma | Angnardo L et al. (2021) | No full text |
| Expression of Maspin and Ezrin proteins in periocular basal cell carcinoma | Bagheri M et al. (2014) | Missing data |
| Targeting the hedgehog pathway for locally advanced and metastatic basal cell carcinoma | Yin VT and Esmaeli B (2017) | No full text |
| Minimum histological safety margins in periocular basal cell carcinoma | Sun MT et al. (2014) | No ocular BCC |
| Conventional frozen sections in periocular basal-cell carcinoma: a review of 236 cases | Glatt HJ et al. (1992) | No full text |
| Collision tumor | Braunstein J et al. (2018) | German |
| Periocular skin cancer: diagnosis and management | Moran JM and Phelps PO (2020) | Review |
| Vismodegib therapy for periocular basal cell carcinoma | Keserü M et al. (2017) | German |
| What's new in eyelid tumors | Silverman N and Shinder R (2017) | Review |
| Redness and periocular pain in a patient with metastatic renal cell carcinoma | Akella M et al. (2021) | No ocular BCC |
| Basal cell carcinosarcoma of the eyelid with osteosarcomatous transformation | Heathcote JG et al. (2021) | Carcinosarcoma |
| Punch biopsy in the management of periocular basal cell carcinomas | Chatterjee S et al. (2004) | Missing data |
| Periocular basal cell carcinoma arising in a site of previous trauma | Keyhani K et al. (2007) | No full text |
| Sebaceous carcinoma of the eyelid | Prieto-Granada C and Rodriguez-Waitkus P (2016) | Sebaceous carcinoma |
| Linear basal cell carcinoma: a distinct clinical entity | Mavrikakis I et al. (2006) | Review |
| Imaging of periocular basal cell carcinoma using en face optical coherence tomography: a pilot study | Khandwala M et al. (2010) | No ocular BCC |
| Risk factors for orbital exenteration in periocular basal cell carcinoma | Shinder R (2012) | Missing data |
| Advances in immunotherapy and periocular malignancy | Habib LA et al. (2019) | Review |
| Clinicopathological factors influencing the number of stages of Mohs surgery for basal cell carcinoma | Calvão J et al. (2020) | Missing data |
| Neglected basal cell carcinoma in a schizophrenic patient | Shah HA et al. (2008) | No full text |
| Basal cell carcinoma of the eyelids | Prabhakaran VC et al. (2007) | Review |
| The role of Mohs excision in periocular basal cell carcinoma | Barnes EA et al. (2006) | Missing data |
| Re: “Imiquimod 5% cream for the treatment of periocular basal cell carcinoma” | Pakdel F and Kashkouli MB (2011) | Missing data |
| Recurrence rates following surgical excision of periocular basal cell carcinomas: systematic review and meta-analysis | Phan K et al. (2020) | Review |
| Sebaceous carcinoma: a review of the scientific literature | Knackstedt T and Samie FH (2017) | Review |
| Demographics of basal cell carcinoma and its surgical management | Afridi RA et al. (2012) | No full text |
| Topical imiquimod 5% as an alternative therapy in periocular basal cell carcinoma in two patients with surgical contraindication | Costales-Álvarez C et al. (2017) | Spanish |
| Surgical management of periocular cancers: high- and low-risk features drive treatment | Allen RC (2017) | Review |
| Fast-tracking' cancer referrals: application for periocular basal cell carcinoma | Bhatnagar A et al. (2006) | Missing data |
| Basal cell carcinoma in xeroderma pigmentosa: reduced CD1a expression as a sensitive predictor of recurrence | El Hanbuli HM et al. (2023) | No full text |
| Socioeconomic and ethnic disparities in periocular cutaneous malignancies | Broadbent T et al. (2016) | Review |
| Mohs surgery for periocular basal cell carcinomas | Robins P et al. (1985) | Missing data |
| Surgical treatment of eyelid tumors | Varde MA et al. (2018) | German |
| The molecular genetics of eyelid tumors: recent advances and future directions | Milman T and McCormick SA (2013) | Review |
| Periocular reconstruction | Bowman PH et al. (2003) | Review |
| Master/slave optical coherence tomography imaging of eyelid basal cell carcinoma | Chin C et al. (2016) | Missing data |
| Periocular cutaneous malignancies: a review of the literature | Slutsky JB and Jones EC (2012) | Review |
| Orbital exenteration: a 23-year report | Kasaee A et al. (2019) | Missing data |
| Basal cell carcinoma treated with Mohs surgery in Australia I. Experience over 10 years | Leibovitch I et al. (2005) | Missing data |
| The impact of COVID-19 on the progression and management of periocular basal cell carcinomas | Garala P and Ahluwalia H (2023) | No full text |
| Medial canthal tophus | Jordan DR et al. (2008) | Tophus |
| Linear basal cell carcinoma: a distinct condition? | Al-Niaimi F and Lyon CC (2011) | Review |
| Review of patients with basal cell nevus syndrome | Taylor SF et al. (2006) | No ocular BCC |
| Mohs micrographic surgery for periocular skin cancer: a single-institution experience | Shi K et al. (2023) | No full text |
| Recurrence following globe sparing excision for basal cell carcinoma with anterior orbital invasion | Tong JY et al. (2023) | No full text |
| Targeting EGFR and sonic hedgehog pathways for locally advanced eyelid and periocular carcinomas | Yin VT et al. (2014) | Review |
| Surgical management of periocular basal cell carcinoma using frozen section control and immediate plastic reconstruction—indications and methods in 106 patients | Holbach LM et al. (1998) | German |
| Topical treatment of periocular basal cell epithelioma with solcoderm | Azizi E et al. (1984) | Missing data |
| Personalized medicine in the treatment of periocular tumors: targeted treatment and use of immune checkpoint inhibitors | Rokohl AC et al. (2020) | German |
| Periocular metatypical cell carcinoma with scleral infiltration | Malik A et al. (2009) | Basosquamous tumor |
| Eyelid complications associated with surgery for periocular cutaneous malignancies | Carniciu AL et al. (2020) | Review |
| Periocular basal cell carcinoma in young adults | Linberg JV (1985) | No full text |
| Periocular microcystic adnexal carcinoma: management and outcome with Mohs' micrographic surgery | Leibovitch I et al. (2006) | Adnexal carcinoma |
| Radiotherapy in periocular cutaneous malignancies: a retrospective study | Lazarevic D et al. (2019) | Missing data |
| Mohs' surgery of periocular basal cell carcinoma using formalin-fixed sections and delayed closure | Skaria AM and Salomon D (1999) | No full text |
| Cutaneous SCC with orbital invasion: case series | Nägeli M et al. (2022) | SCC |
| Periocular skin cancer in solid organ transplant recipients | Perry JD et al. (2016) | Missing data |
| The best of the best: a review of select oculoplastic case series published in 2015 | Temnogorod J and Shinder R (2017) | No ocular BCC |
| Risk factors for canalicular injury after Mohs micrographic surgery | Campbell EH et al. (2022) | No full text |
| Bilateral primary Merkel cell carcinoma of the upper lid misdiagnosed as basal cell carcinoma | Thakur S et al. (2008) | Merkel cell carcinoma |
| Review of targeted therapies for periocular tumors | Mehta VJ et al. (2017) | Review |
| Multicentred international review of orbital exenteration and reconstruction in oculoplastic and orbit practice | Zhang Z et al. (2018) | Missing data |
| Diagnostic utility of adipophilin immunostain in periocular carcinomas | Milman T et al. (2014) | Missing data |
| Laterality of periocular basal cell carcinomas in relation to driving practices in Philadelphia, PA, U.S.A | Schrack KE and Youssef OH (2007) | No ocular BCC |
| Glasses: hiding or causing skin cancer? | Zhang Z et al. (2016) | No ocular BCC |
| Orange palpebral spots: a case presentation | Sangha MS et al. (2022) | No ocular BCC |
| Undiagnosed squamous cell carcinoma of the forehead presenting as a Tolosa–Hunt syndrome | Nieto Enriquez J et al. (2009) | SCC |
| Excision and delayed reconstruction with paraffin section histopathological analysis for periocular sebaceous carcinoma | While B et al. (2014) | Sebaceous carcinoma |
| Sebaceous gland carcinoma of the eyelid | Wali UK and Al-Mujaini A (2010) | Sebaceous carcinoma |
| Extremely advanced basal cell carcinoma in periocular region: a case report | Kecik T et al. (2000) | Polish |
| Imiquimod-side effects in the treatment of periocular skin cancers: a review of the literature | Avallone G et al. (2022) | Review |
| Pathology of the eyelid in elderly patients | Thomas L and Dalle S (2006) | French |
| Clinical and histologic features of 141 primary basal cell carcinomas of the periocular region and their rate of recurrence after surgical excision | Spraul CW et al. (2000) | German |
| Periocular tumors | Salasche SJ et al. (1992) | Review |
| Lines under the eyes: a large prospective case series of linear basal cell carcinomas | Rutkowski D et al. (2023) | No full text |
| A 5-year review of 1220 malignant periocular tumours in an English county | Lin Z et al. (2023) | No full text |
| The use of HCT and/or ACE inhibitors significantly increases the risk of non-melanotic skin cancer in the periocular region | Mehlan J et al. (2022) | Missing data |
| Microcystic adnexal carcinoma of the orbit mimicking pagetoid sebaceous gland carcinoma | Mukherjee B et al. (2018) | Adnexal carcinoma |
| Periocular cutaneous sarcoid: case series and review of the literature | Rajput R et al. (2019) | Periocular cutaneous sarcoid |
| The management of solitary trichoepithelioma versus basal cell carcinoma | Votruba M et al. (1998) | Trichoepithelioma |
| Scalpel excision of basal cell carcinomas | Bart RS et al. (1978) | No full text |
| Transorbital drillout to the cavernous sinus: an approach for squamous cell carcinoma with perineural spread. Illustrative cases | Tong JY et al. (2023) | SCC |
| Laterality of periocular basal cell carcinomas in relation to driving practices in Scotland, United Kingdom | Lim LT et al. (2011) | Missing data |
| Repair of eyelid and periocular soft tissue defects with Pacman flap | Li G et al. (2021) | Chinese |
| Non-surgical treatments of primary, non-melanoma eyelid malignancies: a review | Murchison AP et al. (2011) | Review |
| Fractional cryosurgery for skin cancer | Gonçalves JC (2009) | Missing data |
| Orbital exenteration for advanced periocular adnexal malignancies: curative versus palliative surgical intent | Malik M et al. (2022) | No full text |
| Does chronic exposure to lacrimal fluid predispose for periorbital basal cell carcinoma? | Livaoğlu M and Bektaş D (2008) | No ocular BCC |
| CO_2_ laser and photodynamic therapy: study of efficacy in periocular BCC | Mercuri SR et al. (2018) | Missing data |
| Cumulative experience with Solcoderm in the treatment of basal cell epithelioma | Schewach-Millet M et al. (1984) | Missing data |
| Management and reconstruction of periocular malignancies | Kroll DM (2007) | No ocular BCC |
| Predictors of recurrent basalioma of the eyelids and periorbital region | Zimmermann AC and Klauss V (2001) | German |
| Adipophilin expression in sebaceous tumors and other cutaneous lesions with clear cell histology: an immunohistochemical study of 117 cases | Ostler DA et al. (2010) | Missing data |
| The anatomical distribution of non-melanoma skin cancer: a retrospective cohort study of 22 303 Australian cases | Khalid A et al. (2021) | Missing data |
| Globe-sparing surgical treatment for periocular malignancies with anterior orbital invasion: a consecutive case series | Bergmann MJ et al. (2023) | No full text |
| Efficacy, simplicity, and safety of X-ray therapy of basal-cell carcinomas on periocular skin | Gladstein AH (1978) | No full text |
| Re: "Laterality of periocular basal cell carcinomas in relation to driving practices in Philadelphia, PA, U.S.A." | Jones CA and Adams M (2007) | No ocular BCC |
| Upper and lower eyelid blepharoplasty: development of aesthetic periocular plastic surgery | Horch RE and Arkudas A (2011) | German |
| Periocular Mohs reconstruction by lateral canthotomy with inferior cantholysis: a retrospective study | Mori WS et al. (2021) | No full text |
| The role of cytology in the diagnosis of periocular basal cell carcinomas | Barton K et al. (1996) | Missing data |
| Access to intraoperative tumour margin control: a survey of the British Oculoplastic Surgery Society | Oliphant H et al. (2020) | No ocular BCC |
| Sensitivity and specificity of the diagnosis of periocular lesions by oculoplastic surgeons | Hillson TR et al. (1998) | No full text |
| Factitious disease of periocular and facial skin | Ugurlu S et al. (1999) | No ocular BCC |
| Basal-cell carcinomas of the temple | Carruthers JA et al. (1983) | No ocular BCC |
| Basal cell and squamous cell carcinomas of the eyelid in adults under 50 years of age: 13 cases | Mencía Gutiérrez E et al. (2001) | Spanish |
| Epigenetic silencing contributes to frequent loss of the fragile histidine triad tumour suppressor in basal cell carcinomas | Goldberg M et al. (2006) | Missing data |
| Modified cheek advancement flap for medial lower eyelid, nasal sidewall and infraorbital cheek reconstruction: a case series | Albanese G et al. (2020) | No ocular BCC |
| Use of cyanoacrylate glue casting for stabilization of periocular skin grafts and flaps | Jackson C et al. (2017) | No ocular BCC |
| Periocular lentigo maligna treated with imiquimod | O'Neill J et al. (2011) | Lentigo maligna |
| Development of a PCR for the detection and quantification of parasitism by Demodex folliculorum infestation in biopsies of skin neoplasms periocular area | Tenorio-Abreu A et al. (2016) | Spanish |
| Periocular actinic keratosis, keratinocyte carcinomas, and eyeglasses use | Lee KC et al. (2013) | Missing data |
| Clinicopathological report: Periocular tumour of the follicular infundibulum | Hutchinson KW et al. (2001) | No full text |
| Nasal root island flap for reconstruction of glabellar defects | Martínez SA et al. (2015) | No ocular BCC |
| Desmoplastic trichoepithelioma: report of a unique periocular case | Kirzhner M et al. (2012) | Desmoplastic trichoepithelioma |
| The initial rate of tumour response to Vismodegib treatment can predict a complete response outcome for periocular LA-BCC | Tiosano A et al. (2023) | No full text |
| The Australian Mohs database: short-term recipient-site complications in full-thickness skin grafts | Leibovitch I et al. (2006) | Missing data |
| Role of immunohistochemistry in the diagnosis of sebaceous carcinoma: a clinicopathologic and immunohistochemical study | Plaza JA et al. (2015) | Sebaceous carcinoma |
| Atypical fibroxanthoma of the medial canthus: a rare presentation | Rathore D et al. (2013) | Atypical fibroxanthoma |
| Histologic safety margins of periocular BCC | Rumelt S (2010) | No ocular BCC |
| Periocular dermatitis artefacta in a child | Soong TK et al. (2006) | Periocular dermatitis |
| The modified rhomboid transposition flap in periocular reconstruction | Teske SA et al. (1998) | No ocular BCC |
| Surgical solutions for the reconstruction of the lower eyelid: canthotomy and lateral cantholisis for full-thickness reconstruction of the lower eyelid | Tomassini GM et al. (2008) | No full text |
| Effectiveness of temporal island flap pedicled with perforating branch of zygomatic orbital artery to repair the defects after periocular malignant tumor resection | Zhang Q et al. (2023) | Chinese |
| Retrospective analysis of reconstruction techniques after periocular basalioma excision | Elabjer BK et al. (2007) | No full text |
| A predictive model for primary closure lengths in Mohs surgery based on skin cancer type, dimensions, and location | Theroux ZA et al. (2019) | Missing data |
| Frequency of skin cancer and other ultraviolet radiation provoked skin lesions of eyelid and periocular region in the 1981–1992 period | Talan-Hranilović J et al. (1996) | No full text |
| Skin cancer and actinic keratoses | Ruskiewicz J (1998) | No full text |
| Efficacy of incisional vs punch biopsy in the histological diagnosis of periocular skin tumours | Rice JC et al. (2003) | Missing data |
| Reconstructive surgery of the ocular adnexa: modifications and selected techniques | Gordon Cole J (1965) | Surgical techniques |
| Small margin (2 mm) excision of peri-ocular basal cell carcinoma with delayed repair | David DB et al. (1999) | Missing data |
| Imiquimod 5% cream as an adjuvant pre-operative treatment for basal cell carcinoma of the periocular area | Bonilla R et al. (2014) | Missing data |
| Diagnostic utility of adipophilin immunostain in periocular carcinomas | Milman T et al. (2014) | Missing data |
| Periocular skin cancer in solid organ transplant recipients | Perry JD et al. (2016) | Missing data |
| A retrospective analysis of 147 cases of orbital exenteration | Zhao HS et al. (2023) | Chinese |
| 18F-FDG PET/CT and whole-body bone scan findings in Gorlin-Goltz syndrome | Cheon M et al. (2023) | No ocular BCC |
| Yes-associated protein-1 overexpression in ocular surface squamous neoplasia; a potential diagnostic marker and therapeutic target | Julius P et al. (2023) | Ocular surface squamous neoplasia |
| Case report: Utilization of a scleral lens to mitigate exposure keratopathy and associated mental health decline | Whang K and Brocks D (2023) | No ocular BCC |
| Perifolliculitis mimicking basal cell carcinoma | Asodaria P (2023) | No ocular BCC |
| Basaloid adenocarcinoma of the lacrimal sac: a rare entity and approach to management | Huggins AB et al. (2017) | Basaloid adenocarcinoma |
| Clinicopathologic review of epithelial tumors of the lacrimal gland | Chawla B et al. (2013) | Basaloid adenocarcinoma |
